# The role of Pim-1 kinases in inflammatory signaling pathways

**DOI:** 10.1007/s00011-024-01924-2

**Published:** 2024-07-30

**Authors:** Hye Suk Baek, Nacksung Kim, Jong Wook Park, Taeg Kyu Kwon, Shin Kim

**Affiliations:** 1 Department of Immunology, School of Medicine, Keimyung University, Daegu, 42601 Republic of Korea; 2 Department of Pharmacology, Chonnam University, Gwangju, 61469 Republic of Korea; 3 Institute of Medical Science, Keimyung University, Daegu, 42601 Republic of Korea; 4 Institute for Cancer Research, Keimyung University Dongsan Medical Center, Dalseo-gu, Daegu, 42601 Republic of Korea; 5 Center for Forensic Pharmaceutical Science, Keimyung University, Daegu, 42601 Republic of Korea

**Keywords:** Pim-1 kinase, Inflammatory signaling, LPS, TAK1, NLRP3 inflammasome, Macrophage, T cell response

## Abstract

**Objective and design:**

This observational study investigated the regulatory mechanism of Pim-1 in inflammatory signaling pathways.

**Materials:**

THP-1, RAW 264.7, BV2, and Jurkat human T cell lines were used.

**Treatment:**

None.

**Methods:**

Lipopolysaccharide (LPS) was used to induce inflammation, followed by *PIM1* knockdown. Western blot, immunoprecipitation, immunofluorescence, and RT-PCR assays were used to assess the effect of *PIM1* knockdown on LPS-induced inflammation.

**Results:**

*PIM1* knockdown in macrophage-like THP-1 cells suppressed LPS-induced upregulation of pro-inflammatory cytokines, inducible nitric oxide synthase, cyclooxygenase-2, phosphorylated Janus kinase, signal transducer and activator of transcription 3, extracellular signal-regulated kinase, c-Jun N-terminal kinase, p38, and nuclear factor kappa B p65 (NF-κB p65). It also suppressed upregulation of inhibitor of NF-κB kinase α/β and enhanced the nuclear translocation of NF-κB p65. Moreover, it inhibited the upregulation of Nod-like receptor family pyrin domain-containing 3 (NLRP3) and cleavage of caspase-1 induced by co-treatment of LPS with adenosine triphosphate. Additionally, p-transforming growth factor-β-activated kinase 1 (TAK1) interacted with Pim-1. All three members of Pim kinases (Pim-1, Pim-2, and Pim-3) were required for LPS-mediated inflammation in macrophages; however, unlike Pim-1 and Pim-3, Pim-2 functioned as a negative regulator of T cell activity.

**Conclusions:**

Pim-1 interacts with TAK1 in LPS-induced inflammatory responses and is involved in MAPK/NF-κB/NLRP3 signaling pathways. Additionally, considering the negative regulatory role of Pim-2 in T cells, further in-depth studies on their respective functions are needed.

**Supplementary Information:**

The online version contains supplementary material available at 10.1007/s00011-024-01924-2.

## Introduction

Pim-1 kinase is a serine/threonine protein kinase that belongs to the proviral insertion site in Moloney murine leukemia virus (PIM) family [1, 2]. The Pim kinase family comprises three members: Pim-1, Pim-2, and Pim-3 [3]. Each of these members is located on chromosomes 6, X, and 22 in the human genome [1, 3–5], and they play crucial roles in regulating cell survival, proliferation, and motility [2]. Pim-1 and Pim-3 share 71% amino acid homology, while Pim-1 and Pim-2 share 61% amino acid homology [5]. These Pim kinases exhibit high amino acid sequence similarity, suggesting that they can have both overlapping and distinct roles depending on their tissue distribution. [1, 6]. The role of the pro-oncogene PIM has been primarily studied in tumors, such as hematological malignancies, and in several other cell types, including vascular muscle [7], cardiomyocytes [8], and breast cells [9]. Unlike other kinases, PIM does not have a phosphorylation motif and a regulatory domain, and it is constitutively activated when expressed [5, 10]. Thus, these proteins are regulated at the transcriptional, translational, and proteolytic levels [11]. Pim-1 promotes interferon-beta production in the innate immune response involving the Toll-like receptor (TLR) signaling pathway [12]. In addition, PIM1 inhibition improves colitis by reducing the hyperactivity of macrophages along with T helper (Th) 1 and Th17 immune responses in dextran sodium sulfate colitis mouse models [13]. Further, a *Pim1-*targeting siRNA inhibits lipopolysaccharide (LPS)-induced upregulation of interleukin (IL)-1β in RAW 264.7 cells [14]. Pim-1 inhibition suppresses the production of cytokines that cause allergic inflammation in the airways [15]. Pim-1 represents a potential therapeutic target in rheumatoid arthritis (RA) via regulation of RA fibroblast-like synoviocytes (FLS) [16]. These findings suggest that Pim-1 kinase may serve as a potential therapeutic target for inflammatory regulation. However, the mechanisms underlying PIM functions, including inflammation regulation, remain unknown. Inflammation is a biological response of the immune system that can be triggered by various factors, including pathogens, damaged cells, and toxins [17].

TLRs play an important role in the innate immune system by recognizing pathogen-related molecular patterns derived from various microorganisms [18]. The involvement of the innate immune receptor TLRs in inflammatory diseases has been studied [19]. TLRs are involved in chronic inflammation associated with rheumatoid diseases, including RA, systemic lupus erythematosus, gout, and Lyme’s disease [20]. TLR signaling involves the recruitment of specific adaptor molecules and engages several intracellular proteins, such as nuclear factor kappa-B (NF-κB), mitogen-activated protein kinases (MAPKs), and the NOD-like receptor pyrin domain-containing 3 (NLRP3) inflammasome, which induce inflammatory responses [21–23]. The binding of LPS to TLR4 induces the synthesis and secretion of inflammatory cytokines such as IL-1β, IL-6, and tumor necrosis factor-α (TNF-α) [24]. Various pro-inflammatory cytokines induced by TLR signaling are associated with the Janus kinase (JAK)/signal transducer and activator of transcription (STAT) pathway [25].

Elucidating the pathways associated with these factors and suppressing their excessive production can help develop treatments for inflammatory diseases. Therefore, we investigated the role of Pim-1 kinase in the LPS-mediated inflammatory signaling.

## Materials and methods

### Reagents

LPS (From *Escherichia coli* serotype 0111:B4) was purchased from Sigma-Aldrich (L391, St. Louis, MO, USA). The pan-PIM kinase inhibitors, PIM447 and AZD1208, were purchased from Selleck Chemicals (#S7985, #S7104, Houston, TX, USA). Transforming growth factor-β-activated kinase 1 (TAK1) inhibitor (5Z)-7-oxozeaenol was purchased from Tocris Bioscience (#3604, Bristol, United Kingdom). Adenosine 5′-triphosphate disodium salt (ATP) and phorbol myristate acetate (PMA) were purchased from Santa Cruz Biotechnology (sc-202040, Santa Cruz, CA, USA) and Sigma-Aldrich (P1585), respectively. Antibodies against MyD88 (#4283), phosphorylated-inhibitor of NF-κB kinase α/β (p-IKKα/β, Ser176/180, #2697), IKKα (#11930), p-NF-κB p65 (Ser536, #3033), p-Bad (Ser136, #9265), extracellular signal-regulated kinase (ERK), p-ERK (Thr202/Tyr204 #9101), inducible nitric oxide synthase (iNOS, #13120), NLRP3 (#15101), p-JAK1 (Tyr1034/1035, #3331), p-STAT3 (Tyr705, #9145), p-JNK (Thr183/Tyr185, #9251), JNK (#9252), and p-TAK1 (Ser412, #9339) were purchased from Cell Signaling Technology (Beverly, MD, USA). Anti-IL-1β antibodies were purchased from Novus Biologicals (NB600-633, Centennial, CO, USA). Antibodies against TLR4 (sc-293072), speck-like protein containing a CARD (ASC, sc-22514-R), caspase-1 (sc-56036), Pim-1 (sc-13513), Pim-3 (sc-293237), IL-6 (sc-130326), nuclear factor of activated T-cells 1 (NFATc-1, sc-7294), JAK1 (sc-1677), STAT3 (sc-8019), p-p38 (sc-7972), p38 (sc-7972), IL-2 (sc-133118) and TAK1 along with anti-horse IgG-horseradish peroxidase (HRP), anti-mouse IgG-HRP, normal mouse IgG (sc-2025) and anti-rabbit IgG-HRP antibodies were purchased from Santa Cruz Biotechnology. Pim-2 (ab129057), TNF alpha (ab6671), Anti-CD3(BE0231) and anti-CD28 (BE0291) were purchased from Bio X cell, inc. (Lebanon, NH, USA). Anti-β actin antibodies and anti-COX-2 (SAB4200576) were purchased from Sigma-Aldrich. The WelCount™ Cell Proliferation Assay Kit ([2,3-Bis(2-methoxy-4-nitro-5-sulfophenyl)-2 H-tetrazolium-5-carbox anilide]) was purchased from Welgene (Gyeongsan, South Korea).

### Constructs

The deletion mutant form of Pim-1 kinase, pMX-dominant negative form of Pim-1 (aa 81–313) (Pim-1 DN), was constructed by deleting the N-terminal region of Pim-1, which contains a kinase domain [26]. A point mutant form of Pim-1, pMX-Pim-1-K67M-Flag (Pim-1 K67M) [27], was generated using the QuickChange method of site-directed mutagenesis (Stratagene, La Jolla, CA, USA).

### Cell lines and culture

All cell lines used in this study were continuously cultured and used at the end of the experiment. THP-1 human monocytic leukemia cell line, RAW 264.7 murine macrophage cell line, BV2 murine microglial cell line, and Jurkat human T cell line were used in the study. THP-1 and RAW 264.7 cells were purchased from the Korea Cell Line Bank (Seoul, South Korea) and cultured in the Roswell Park Memorial Institute medium (Welgene. Gyeongsan, Korea) and Dulbecco’s modified Eagle’s medium (Gibco, Grand Island, NY, USA). Prior to investigating the inflammatory signaling pathway in macrophages, THP-1 cells were differentiated into macrophage-like THP-1 cells via PMA treatment. Briefly, plates were seeded with an appropriate number of cells for each experiment and incubated for 24 h with 100 nM PMA diluted in the medium. A 1% antibacterial–antifungal solution (Gibco) and 10% fetal bovine serum (Gibco) were added to the culture medium. The humidity and temperature were maintained at 95% and 37 °C, respectively, with a continuous supply of 5% CO_2_. The cells were subcultured once or twice a week.

### Small-interfering RNA transfection

THP-1 cells were seeded on 12-well plates, differentiated via PMA treatment, and transfected with *PIM1*, *PIM2*, and *PIM3* small-interfering RNA (siRNA) (Life Technologies, Gaithersburg, MD, USA), and control siRNA using transfection reagent (sc-37007, Santa Cruz Biotechnology). All transfections were performed using Lipofectamine™ RNAiMAX (Life Technologies). THP-1 cells were transfected with 30–45 nM siRNA per well. After 72 h, the cells were used for further experiments. RAW 264.7 and BV2 cells were seeded on 12-well plates at a density of 3 × 10^5^ cells/well. Plasmid DNA was mixed with Lipofectamine 2000 reagent (Life Technologies) and added to the cells according to the manufacturer’s protocol.

### Western blotting

THP-1 cells were plated at densities of 1 × 10^6^ cells/well and 2 × 10^6^ cells/well in 100 and 60 mm dishes, respectively. Subsequently, total proteins were extracted from the processed cells via lysis in radioimmunoprecipitation (RIPA) assay buffer (Cell Signaling Technology). Cytoplasmic and nuclear protein were isolated using extraction reagents (NE-PER™, Thermo Scientific, Rockford, IL, USA). Protein concentrations were measured using a bicinchoninic acid assay kit (Thermo Fisher Scientific, Wilmington, NC, USA). Cell culture medium supernatants were precipitated using trichloroacetic acid (TCA), as described previously [28]. Briefly, one volume of 100% TCA stock was added to four volumes of the protein sample. The mixture was incubated for 10 min at 4 °C, spin-downed, and the supernatant was removed while leaving the protein pellet intact. The precipitated pellets were washed with 200 µL cold acetone. The pellets were dried by placing the tube in a 95 °C heat block for 5–10 min to remove acetone. For sodium dodecyl sulfate (SDS)-polyacrylamide gel electrophoresis, the sample was suspended in 20 µL 2× or 4× sample buffer (with or without β-mercaptoethanol) and boiled for 10 min at 95 °C before loading onto a polyacrylamide gel. Equal amounts of protein were electrophoresed on 10% SDS-polyacrylamide gels and transferred to nitrocellulose membranes. The membranes were blocked with 5% skim-milk for 1 h and then incubated for 24 h with appropriately diluted primary antibodies against specific target proteins. Subsequently, the membranes were washed with Tris-buffered saline/Tween buffer and then incubated at room temperature for 1 h with HRP-conjugated anti-IgG secondary antibodies. Lastly, enhanced chemiluminescence was used to detect the protein bands, and the signal strength was measured using a chemiluminescence imaging system (Fusion Fx7; Vilber Lourmat, Collégien, France). Protein bands were quantified using Image J software program. β-actin was used as the internal control for Western blot analysis.

### Immunoprecipitation

The cells were harvested and washed with cold PBS, after which proteins were extracted using a lysis buffer (0.5% NP-40, 0.5 M Tris (pH 7.4), 5 M NaCl, 0.5 M EDTA, and 0.5 M MgCl_2_). The concentration of proteins in the whole-cell lysates was measured, and all samples were incubated overnight with primary antibodies at 4 °C. The cell lysates were then incubated with protein G-agarose (sc-2002, Santa Cruz Biotechnology) for 2 h. The beads were washed in lysis buffer and boiled for 10 min. Immunoprecipitation was confirmed via Western blotting. Flag-tagged Pim-1 DN, Pim-1 WT, and Pim-1-K67M were immunoprecipitated by anti-FLAG antibody (F1804, Sigma-Aldrich).

### RNA isolation and reverse transcription-quantitative polymerase chain reaction

Total RNA was extracted from each sample using TRIzol solution (Invitrogen, San Diego, CA, USA) according to the manufacturer’s protocol. Subsequently, 1 µg of the extracted and quantified RNA was reverse transcribed using deoxynucleoside triphosphate, buffer, dithiothreitol, RNase inhibitor, and SuperScript II reverse transcriptase. The synthesized cDNA was used for reverse transcription-quantitative polymerase chain reaction performed using specific primers. The primers used in the study are listed in supplementary Table 1.

### Immunofluorescence staining

THP-1 cells (1 × 10^3^ cells) were cultured in eight-chamber glass slides for 24 h, differentiated via PMA treatment, and then stimulated with LPS for 6 h. The cells were washed with PBS, fixed with 4% formaldehyde, permeabilized using 0.2% Triton X-100 in PBS, and incubated with bovine serum albumin for 1 h to block nonspecific binding. Subsequently, the cells were incubated with primary antibodies at 4 °C for 24 h and then with fluorescein isothiocyanate-conjugated goat anti-mouse IgG (Thermo Fisher Scientific, Wilmington, NC, USA) and 4′,6-diamidino-2-phenylindole for nuclear staining (Invitrogen), after which the stained cells were examined under a fluorescence microscope.

### Statistical analysis

Differences between the groups were analyzed using the Student’s two-tailed *t*-test. Experimental results were analyzed using GraphPad Prism 5.0 (GraphPad Software, San Diego, CA, USA). One-way analysis of variance was performed for comparison between groups, and the significance between the control and experimental groups was analyzed at the *p* < 0.05 level using the Tukey’s multiple composition test (* *p* < 0.05, ** *p* < 0.01, # *p* < 0.001).

## Results

### LPS upregulates Pim-1 and pro-inflammatory cytokines in macrophage-like THP-1 cells

**Fig. 1:**
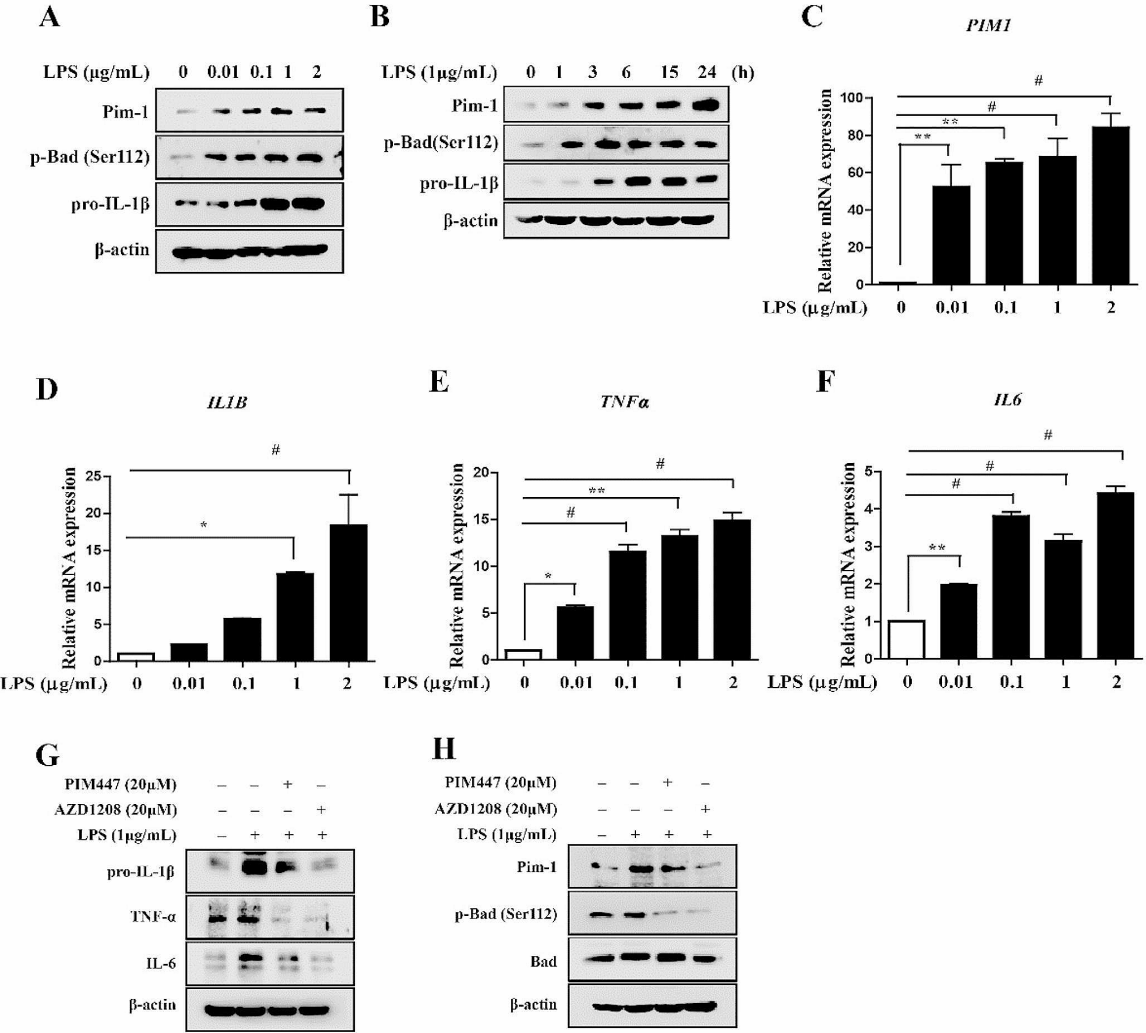
The expression levels of Pim-1 and pro-inflammatory cytokines in LPS-stimulated macrophage-like THP-1 cells. (**A**) Cells were stimulated with LPS (0.01, 0.1, 1, and 2 µg/mL) for 6 h. Whole cell lysates were isolated and used to measure the protein expression levels of Pim-1, p-Bad, and pro-IL-1β by Western blotting. (**B**) Cells were stimulated with LPS (1 µg/mL) for the indicated time points. Whole cell lysates were isolated and used to measure the protein expression levels of Pim-1, p-Bad, and pro-IL-1β by Western blotting. (**C**-**F**) Cells were stimulated with LPS (0.01, 0.1, 1, and 2 µg/mL) for 6 h. Total RNA was extracted, and used to evaluate the mRNA expression levels of *PIM1*, *IL1B*, *TNFα*, and *IL6* by real-time qPCR (* *p* < 0.05, ** *p* < 0.01, # *p* < 0.001). (**G**) THP-1 cells were differentiated into macrophages using 100 nM PMA for 24 h, then the cells were stimulated with LPS (1 µg/mL) for 6 h after pre-treatment with PIM447 (20 µM) and AZD1208 (20 µM) for 1 h. Whole cell lysates were isolated and used to measure the protein expression levels of IL-1β, TNF-α and IL-6 by Western blotting. (**H**) Whole cell lysates were isolated and used to measure the protein expression levels of Pim-1, p-Bad (Ser112), and Bad by Western blotting

To investigate the role of Pim-1 in the TLR4-mediated inflammatory response, we stimulated TLR4 present on macrophage-like THP-1 cells using LPS. LPS treatment upregulated Pim-1, p-BCL2-associated agonist of cell death (Bad) (Ser112), and pro-IL-1β in a dose- and time-dependent manner (Fig. 1A, B). It has been reported that the concentration and time period of LPS treatment can influence macrophage M1/M2 polarization [29–31]. Consequently, we evaluated macrophage polarization under our experimental conditions. The results demonstrated that LPS treatment induced M1-type macrophage polarization (supplementary Fig. 3) and increased the mRNA expression levels of *PIM1*, *IL1B*, *TNFα*, and *IL6* in a dose-dependent manner (Fig. 1C–F). To determine whether Pim kinases play important roles in TLR4-mediated inflammatory responses in macrophage-like THP-1 cells, we treated cells with two pan-PIM kinase inhibitors (PIM447 and AZD1208). Both inhibitors suppressed the LPS-induced upregulation of pro-IL-1β, TNF-α, IL-6, Pim-1, and p-Bad (Ser112) in macrophage-like THP-1 cells (Fig. 1G, H). Therefore, the upregulation of Pim-1 is involved in the LPS-mediated inflammatory response.

### PIM1 knockdown attenuates the LPS-mediated upregulation of pro-inflammatory cytokines in macrophage-like THP-1 cells

**Fig. 2:**
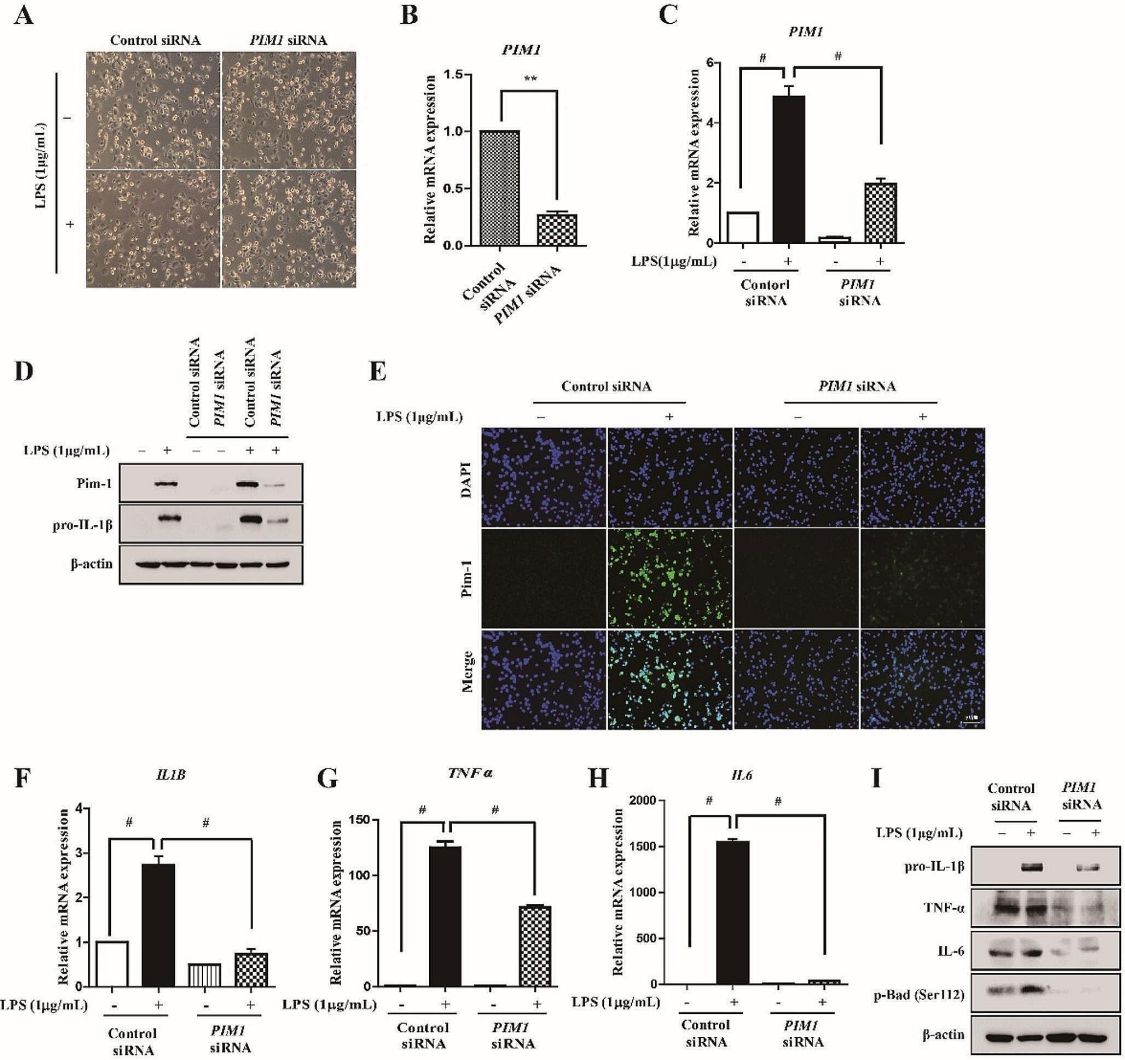
The effect of *PIM1* knockdown in LPS-mediated inflammatory signals of macrophage-like THP-1 cells. THP-1 cells transfected with control siRNA or *PIM1* siRNA for 72 h and then stimulated with LPS (1 µg/mL) for 6 h. (**A**) The effects of *PIM1* siRNA on cellular morphological changes were observed by microscopy (×200). (**B**, **C**) Total RNA was extracted, and used to evaluate the mRNA expression levels of *PIM1* by real-time qPCR, (** *p* < 0.01, # *p* < 0.001). (**D**) Whole cell lysates were isolated and used to measure the protein expression levels of Pim-1 and pro-IL-1β by Western blotting. (**E**) Cells were stained with antibodies to Pim-1 (green) and DAPI (blue) and captured at ×200 using fluorescence microscope (scale bar = 50 μm). (**F**-**H**) Total RNA was extracted, and used to evaluate the mRNA expression levels of pro-inflammatory cytokines (*IL1B*, *TNFα*, and *IL6*) (# *p* < 0.001). (**I**) Whole cell lysates were isolated and used to measure the protein expression levels of pro-IL-1β, IL-6, TNF-α, and p-Bad (Ser112) by Western blotting

Since Pim kinases are constitutively active [32], *PIM1* knockdown using siRNA transfection allows the regulation of function, similar to treatment with a selective Pim-1 inhibitor. To determine whether Pim-1 regulates pro-inflammatory cytokine production, human *PIM1*-specific siRNA was used. Phase-contrast microscopy revealed no noticeable change in the external morphology of macrophage-like THP-1 cells when transfected with either control or *PIM1* siRNA, with or without LPS treatment (Fig. 2A). *PIM1* knockdown significantly downregulated *PIM1* in macrophage-like THP-1 cells (Fig. 2B–E). Next, we investigated the effect of *PIM1* knockdown on pro-inflammatory cytokines. *PIM1* knockdown attenuated the LPS-mediated upregulation of *IL1B*, *TNFα*, and *IL6* in macrophage-like THP-1 cells (Fig. 2F–H). In addition, *PIM1* knockdown attenuated the LPS-mediated upregulation of pro-IL-1β, TNF-α, and IL-6 protein levels in macrophage-like THP-1 cells. These results suggested that Pim-1 kinase is involved in the LPS-mediated upregulation of various pro-inflammatory cytokines at the transcriptional level.

### PIM1 knockdown attenuates LPS-mediated upregulation of iNOS and COX-2, phosphorylation of MAPKs, and activation of NF-κB in macrophage-like THP-1 cells

**Fig. 3:**
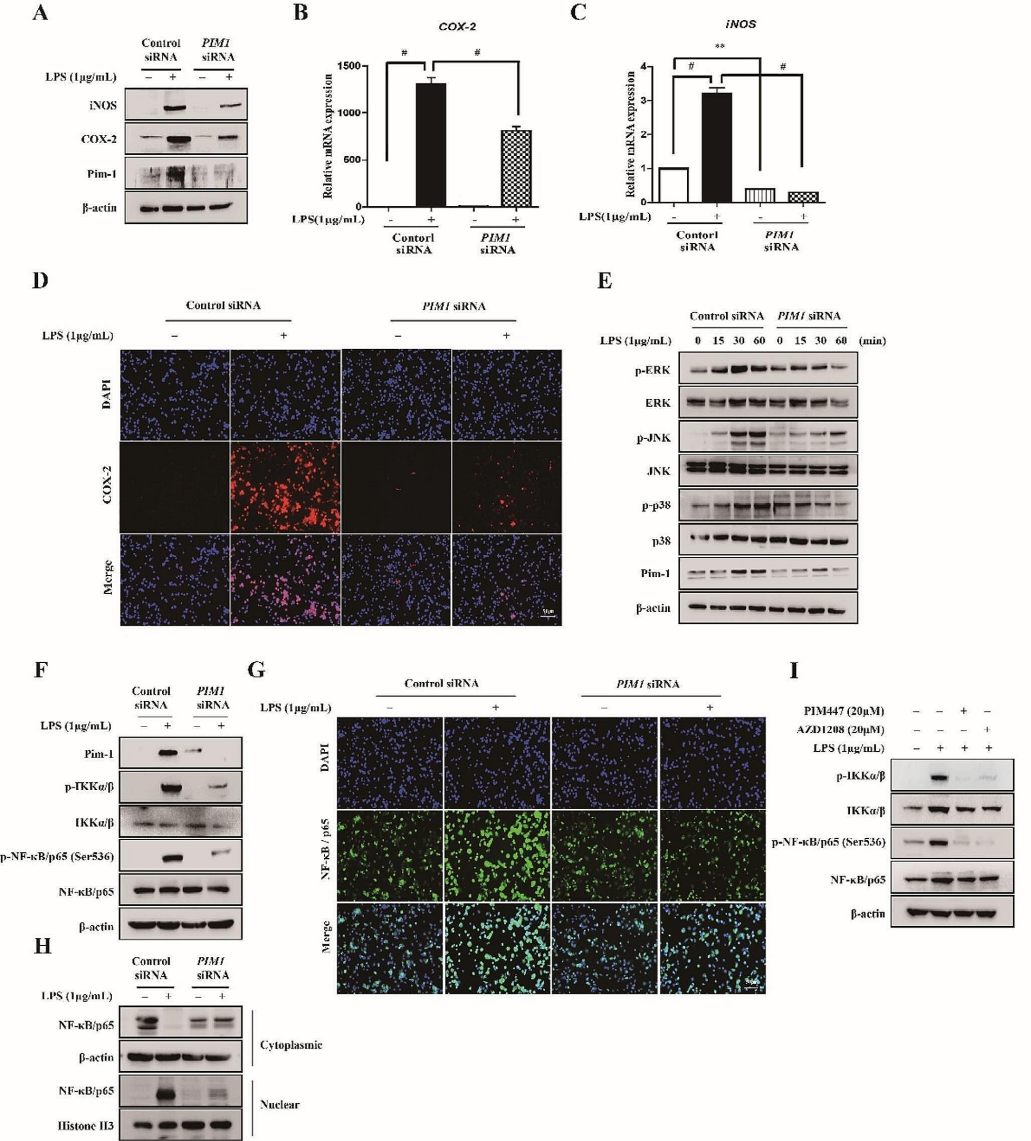
The effect of *PIM1* knockdown in LPS-induced iNOS, COX-2, MAPKs and NF-κB in macrophage-like THP-1 cells. (**A**) Whole cell lysates were isolated and used to measure the protein expression levels of iNOS and COX-2 by Western blotting. (**B**, **C**) Total RNA was extracted, and used to evaluate the mRNA expression levels of iNOS and COX-2, respectively (** *p* < 0.01, # *p* < 0.001). (**D**) Cells were stained with antibodies to COX-2 (red) and DAPI (blue) and captured at ×200 using fluorescence microscope (scale bar = 50 μm). (**E**) Cells were stimulated with LPS (1 µg/mL) for the indicated time points. Whole cell lysates were isolated and used to measure the protein expression levels of p-ERK, ERK, p-JNK, JNK, p-p38, and p38 by Western blot analysis. (**F**) Whole cell lysates were isolated and used to measure the protein expression levels of p-IKKα/β and p-NF-κB p65 by Western blotting. (**G**) Cells were stained with antibodies to NF-κB p65 (green) and DAPI (blue) and captured at ×200 using fluorescence microscope (scale bar = 50 μm). (**H**) Cytoplasmic and nuclear proteins were extracted and assayed by Western blot analysis using anti-NF-κB p65 antibody. The expression levels of actin and histone 3 were used as loading controls. (**I**) THP-1 cells were differentiated into macrophages using 100 nM PMA for 24 h, then the cells were stimulated with LPS (1 µg/mL) for 6 h after pre-treatment with PIM447 (20 µM) and AZD1208 (20 µM) for 1 h. Whole cell lysates were isolated and used to measure the protein expression levels of p-IKKα/β and p-NF-κB p65 by Western blotting

We determined whether Pim-1 activation by LPS is involved in the TLR4-mediated regulation of the inflammation-related enzymes iNOS and COX-2, which induce inflammatory responses. *PIM1* knockdown inhibited the LPS-induced upregulation of iNOS and COX-2 at the protein and mRNA levels (Fig. 3A–C). Moreover, *PIM1* knockdown suppressed the LPS-induced increase of COX-2 protein level in immunofluorescence analysis (Fig. 3D). LPS treatment upregulated phosphorylated IKKα/β, NF-κB p65, ERK, JNK, and p38 in macrophage-like THP-1 cells, whereas *PIM1* knockdown inhibited the LPS-mediated phosphorylation (Fig. 3E, F). *PIM1* knockdown also suppressed the nuclear translocation of NF-κB p65, based on fluorescence microscopy analysis and isolation of nuclear and cytosolic proteins, which were subjected to Western blot analysis in macrophage-like THP-1 cells (Fig. 3G, H). Similar to the results of *PIM1* knockdown, treatment with the two pan-PIM kinase inhibitors inhibited the LPS-mediated activation of NF-κB in macrophage-like THP-1 cells. Therefore, Pim-1 kinase is involved in various LPS-mediated inflammatory signaling pathways.

### PIM1 knockdown attenuates LPS-mediated NLRP3 inflammasome activation in macrophage-like THP-1 cells

**Fig. 4:**
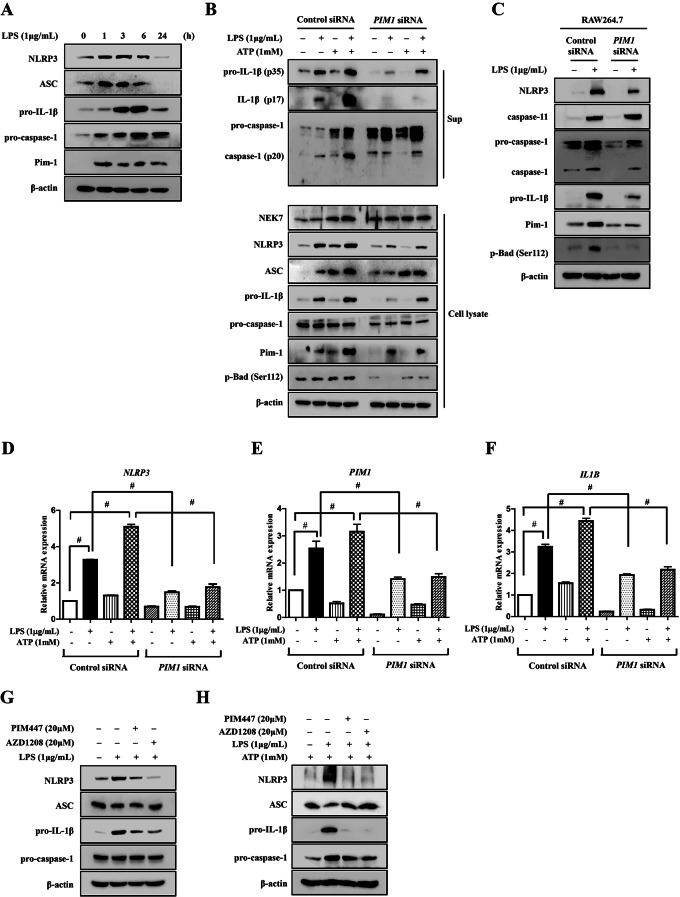
The effect of *PIM1* knockdown in LPS-mediated activation of NLRP3 inflammasome in macrophage-like THP-1 cells. (**A**) Cells were stimulated with LPS (1 µg/mL) for the indicated time points. Whole cell lysates were isolated and used to measure the protein expression levels of NLRP3, ASC, pro-IL-1β, and pro-caspase-1 by Western blotting. (**B**) Cells were stimulated with LPS (1 µg/mL) and/or ATP (1 mM) for 6 h. Cell lysate (Lysate) and media supernatant (Sup) were isolated and used to measure the protein expression levels of pro-IL-1β, IL-1β, pro-caspase-1, and caspase-1 for the Sup as well as NLRP3, ASC, pro-caspase-1, and pro-IL-1β for the Lysate by Western blot analysis. (**C**) RAW 264.7 cells transfected with control siRNA or *Pim-1* siRNA for 72 h and then stimulated with LPS (500 ng/mL) for 6 h. Whole cell lysates were isolated and used to measure the protein expression levels of NLRP3, caspase-11, pro-IL-1β, pro-caspase-1, and caspase-1 by Western blotting. (**D**-**F**) Total RNA was extracted, and used to evaluate the mRNA expression levels of *NLRP3*, *IL1B*, and *PIM1*, respectively (# *p* < 0.001). (**G**,**H**) Cells were differentiated into macrophages using 100 nM PMA for 24 h, then the cells were stimulated with LPS (1 µg/mL) with/without ATP (1mM) for 6 h after pretreatment with PIM447 (20 µM) and AZD1208 (20 µM) for 1 h. Whole cell lysates were isolated and used to measure the protein expression levels of NLRP3, ASC, pro-IL-1β, and pro-Caspase-1 by Western blotting

We investigated the role of Pim-1 in activation of the NLRP3 inflammasome, which is an important mechanism in inflammatory responses. LPS treatment upregulated Pim-1 in macrophage-like THP-1 cells. Moreover, the protein expression levels of NLRP3, ASC, pro-IL-1β, and pro-caspase-1 were increased by LPS treatment (Fig. 4A). *PIM1* knockdown inhibited the secretion of pro-IL-1β, IL-1β, and cleaved caspase-1 into the cell culture media and inhibited the protein expression levels of NLRP3, pro-IL-1β, and pro-caspase-1 in macrophage-like THP-1 cells co-stimulated with LPS and ATP (Fig. 4B). Additionally, *PIM1* knockdown significantly attenuated the upregulation of *PIM1*, *NLRP3*, and *IL1B* induced by LPS and by co-treatment with LPS and ATP (Fig. 4D–F). Similar to the results obtained for macrophage-like THP-1 cells, *Pim1* knockdown inhibited the LPS-induced upregulation of NLRP3, pro-caspase-1, cleaved caspase-1, pro-IL-1β, Pim-1, and p-Bad (Ser112) at the protein level in RAW 264.7 cells (Fig. 4C). However, the expression of caspase-11, which promotes NLRP3 inflammasome activation and increases pro-IL-1β levels [33, 34], was not affected by *Pim1* knockdown (Fig. 4C). Furthermore, treatment with both pan-PIM kinase inhibitors inhibited the LPS-induced upregulation of NLRP3 and Pro-IL-1β protein levels (Fig. 4G, H). Therefore, Pim-1 kinase is involved in the LPS-mediated NLRP3 inflammasome signaling pathways.

### Pim-1 and TAK1 regulate each other’s kinase activity in LPS-mediated inflammatory response of macrophage-like THP-1 cells

**Fig. 5:**
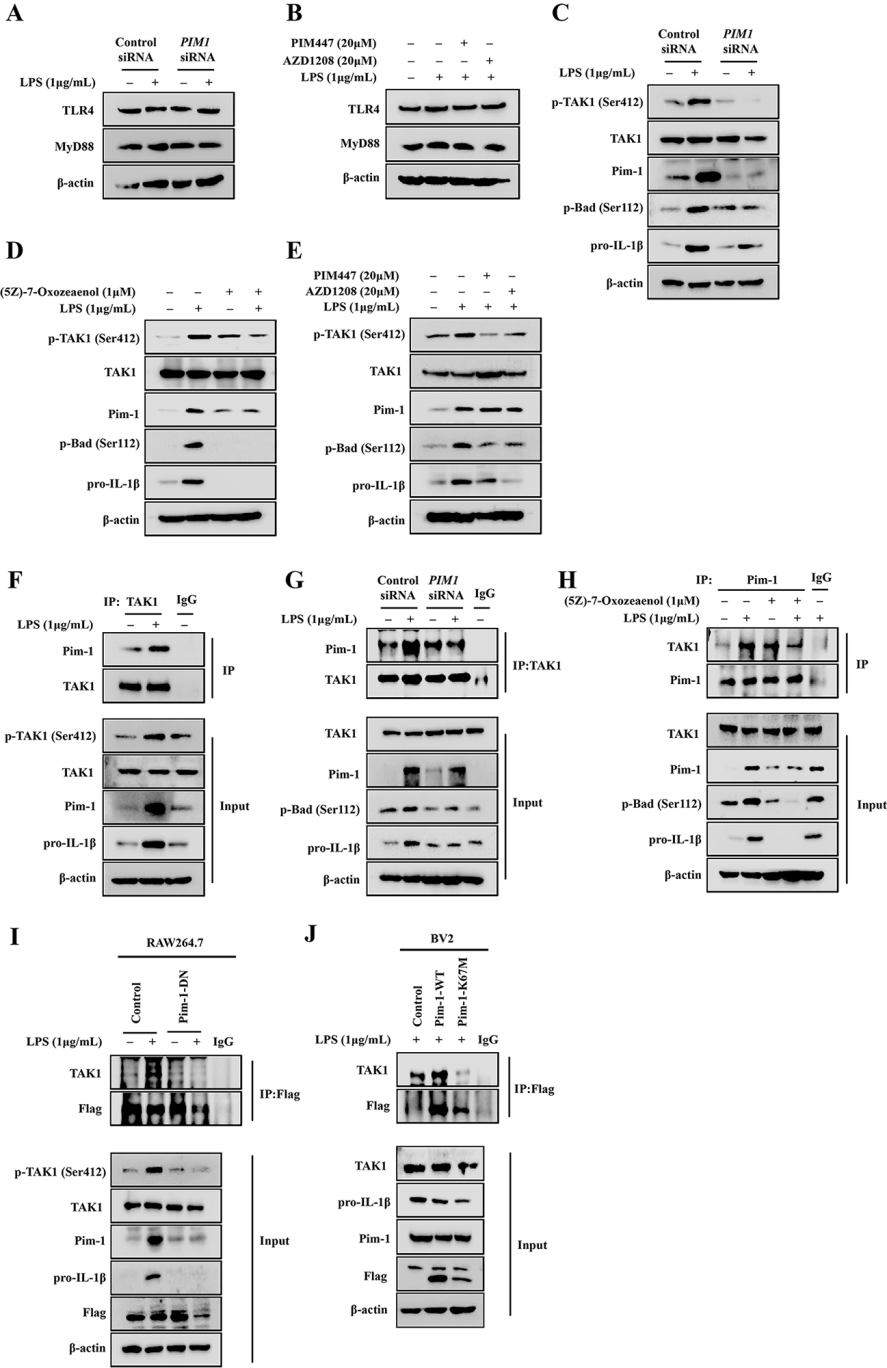
The effect of *PIM1* knockdown in LPS-mediated activation of TAK1 expression in macrophage-like THP-1 cells. (**A**) Whole cell lysates were isolated and used to measure the protein expression levels of TLR4 and MyD88 by Western blotting. (**B**) Cells were stimulated with LPS (1 µg/mL) for 6 h after pre-treatment with PIM447 (20 µM) and AZD1208 (20 µM) for 1 h. Whole cell lysates were isolated and used to measure the protein expression levels of TLR4 and MyD88 by Western blotting. (**C**-**E**) Cells were transfected with control siRNA or *PIM1* siRNA, and pre-treatment with TAK1 inhibitor (5Z)-7-Oxozeaenol (1 µM), or with PIM447 (20 µM) and AZD1208 (20 µM) for 1 h before LPS (1 µg/mL) stimulation. Whole cell lysates were isolated and used to measure the protein expression levels of p-TAK1, TAK1 and pro-IL-1β by Western blotting. Interaction of Pim-1 with TAK1 protein in LPS-induced THP-1 cells. (**F**) THP-1 cells were stimulated with LPS (1 µg/mL) for 6 h, and cell lysates were subjected to immunoprecipitation with TAK1, then the protein expression levels of Pim-1 and TAK1 were detected by Western blotting. (**G**) Cells were transfected with control siRNA or *PIM1* siRNA, and Whole cell lysates were subjected to immunoprecipitation with TAK1, then the protein expression levels of Pim-1 and TAK1 were detected by Western blotting. (**H**) Cells were pre-treatment with 1 µM of TAK1 inhibitor (5Z)-7-Oxozeaenol for 1 h before LPS (1 µg/mL) stimulation. Whole cell lysates were subjected to immunoprecipitation with Pim-1, then the protein expression levels of TAK1 and Pim-1 were detected by Western blotting. (**I**, **J**) RAW 264.7 and BV2 cells were transiently transfected with plasmids expressing pMX-IRES-EGFP-Flag empty vector (control) or pMX-Pim-1 DN, pMX-Pim-1 WT, and pMX-Pim-1 K67M (mutant forms). The cell lysates were subjected to immunoprecipitation with anti-Flag, then the protein expression levels of TAK1 and Flag were detected by Western blotting

Since TAK1 plays an important role in TLR4/myeloid differentiation primary response 88 (MYD88)-mediated inflammatory signaling, we investigated the correlation of Pim-1 and TAK1 in LPS-mediated inflammatory signaling. First, *PIM1* knockdown and pan-PIM kinase inhibitor treatment did not alter the protein expression levels of TLR4 and MyD88 (Fig. 5A, B). *PIM1* knockdown suppressed the LPS-mediated upregulation of p-TAK1 (Ser412), Pim-1, p-Bad (Ser112), and pro-IL-1β protein levels in macrophage-like THP-1 cells (Fig. 5C). In addition, treatment with (5Z)-7-oxozaenol, a TAK1 inhibitor, suppressed the LPS-induced increase in Pim-1, p-Bad (Ser112), and pro-IL-1β protein levels in macrophage-like THP-1 cells (Fig. 5D). The PIM kinase inhibitors suppressed the LPS-induced increase in p-TAK1 (Ser412), p-Bad (Ser112), and pro-IL-1β protein levels in macrophage-like THP-1 cells (Fig. 5E). Therefore, Pim-1 and TAK1 are correlated in LPS-mediated inflammatory signaling pathways.

### The kinase activities of Pim-1 and TAK1 regulate each other via reciprocal binding

Immunoprecipitation analysis was performed to further investigate the interaction between Pim-1 and TAK1. LPS treatment induced the interaction between endogenous Pim-1 and TAK1 in macrophage-like THP-1 cells (Fig. 5F, G). Moreover, both *PIM1* knockdown and (5Z)-7-oxozaenol treatment inhibited the LPS-stimulated interaction between endogenous Pim-1 and TAK1 along with the downregulation of pro-IL-1β in macrophage-like THP-1 cells (Fig. 5G, H). In addition, pim-1-DN suppressed the LPS-induced interaction between endogenous Pim-1 and TAK1, along with the downregulation of pro-IL-1β in RAW 264.7 cells (Fig. 5I). Notably, Pim-1-K67M attenuated the LPS-induced interaction between endogenous Pim-1 and TAK1, along with the downregulation of pro-IL-1β in BV2 cells (Fig. 5J). Therefore, Pim-1 and its interaction with TAK1 play a critical role in the LPS-mediated inflammatory signaling pathways.

### PIM1 knockdown attenuates LPS-mediated activation of the JAK/STAT pathway in macrophage-like THP-1 cells

**Fig. 6:**
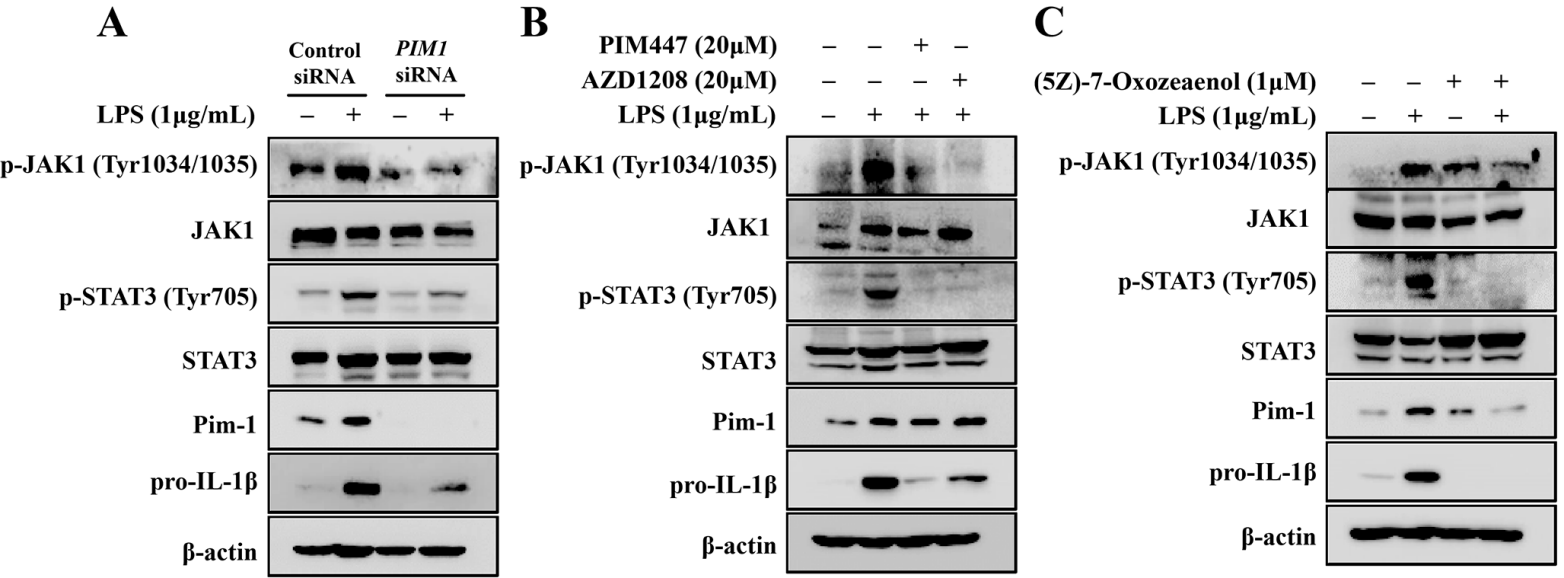
The effect of *PIM1* knockdown in LPS-induced JAK/STAT pathway in macrophage-like THP-1 cells. (**A**) Cells were transfected with control siRNA or *PIM1* siRNA. (**B**) Cells were pre-treatment with TAK1 inhibitor (5Z)-7-Oxozeaenol (1 µM). (**C**) Cells were with PIM447 (20 µM) and AZD1208 (20 µM) for 1 h before LPS (1 µg/mL) stimulation. Whole cell lysates were isolated and used to measure the protein expression levels of p-JAK1, JAK1, p-STAT3, STAT3, and pro-IL-1β by Western blotting

Next, we investigated whether Pim-1 plays an important role in LPS-induced activation of the JAK/STAT pathway. *PIM1* knockdown inhibited LPS-mediated upregulation of p-JAK1 (Tyr1034/1035) and p-STAT (Tyr705) in macrophage-like THP-1 cells (Fig. 6A). Moreover, PIM447, AZD1208, and (5Z)-7-Oxozaenol treatment inhibited the LPS-stimulated upregulation of p-JAK1 (Tyr1-34/1035) and p-STAT (Tyr705) in macrophage-like THP-1 cells (Fig. 6B, C). Therefore, Pim-1 functions as an upstream modulator of JAK1 and STAT3.

### Pim-2 has different functions in the inflammatory response of macrophage-like THP-1 and Jurkat T cells

**Fig. 7:**
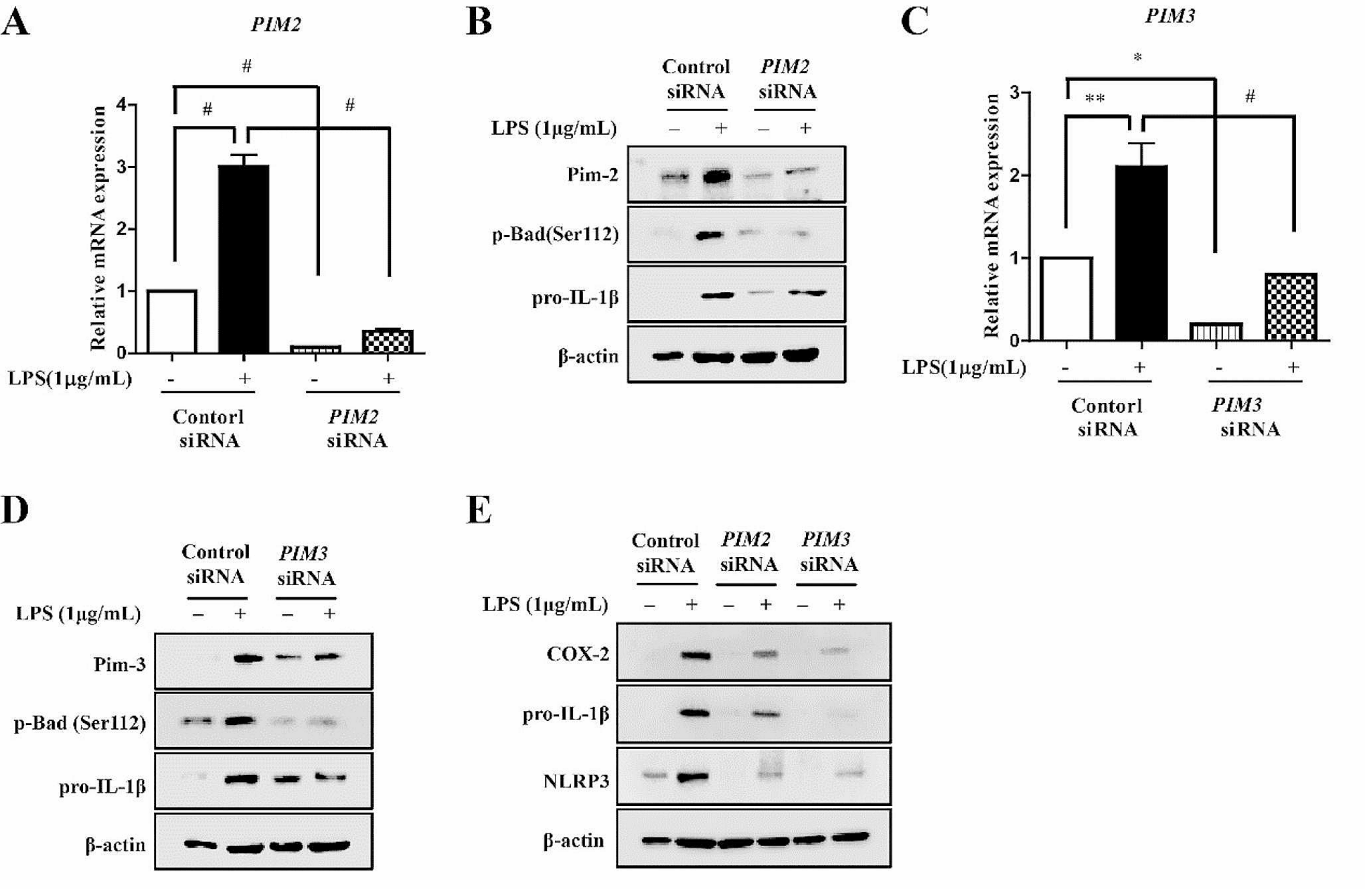
The effect of *PIM2* and *PIM3* kinase knockdown in macrophage-like THP-1. THP-1 cells transfected with control siRNA or *PIM2* siRNA for 72 h and then stimulated with LPS (1 µg/mL) for 6 h. (**A**) Total RNA was extracted, and used to evaluate the mRNA expression levels of *PIM2* (# *p* < 0.001). (**B**) Whole cell lysates were isolated and used to measure the protein expression levels of pro-IL-1β and p-Bad by Western blotting (**C**) THP-1 cells transfected with control siRNA or *PIM3* siRNA for 72 h and then stimulated with LPS (1 µg/mL) for 6 h. Total RNA was extracted, and used to evaluate the mRNA expression levels of *PIM2* by real-time qPCR (* *p* < 0.05, ** *p* < 0.01, # *p* < 0.001). (**D**) Whole cell lysates were isolated and used to measure the protein expression levels of pro-IL-1β and p-Bad by Western blotting (**E**) THP-1 cells transfected with control siRNA or *PIM2*, *3* siRNA for 72 h, respectively. Whole cell lysates were isolated and used to measure the protein expression levels of COX-2, pro-IL-1β and NLPR3 by Western blotting

To determine whether the other isotypes of Pim kinase, including Pim-2 and Pim-3, affect the inflammatory response in different immune cells, human *PIM-2* and *PIM-3*-specific siRNAs were used to transfect cells. Both *PIM2* and *PIM3* knockdown significantly inhibited the LPS-mediated upregulation of *PIM2* and *PIM3* at the protein and mRNA levels in macrophage-like THP-1 cells (Fig. 7A–D). Furthermore, knockdown of both *PIM2* and *PIM3* inhibited the LPS-mediated increase in p-Bad (Ser112) and pro-IL-1β protein levels in macrophage-like THP-1 cells (Fig. 7A, C). Similar to the results of *PIM1* knockdown, knockdown of both *PIM2* and *PIM3* attenuated the LPS-induced increase of COX-2, pro-IL-1β, and NLRP3 protein levels in macrophage-like THP-1 cells (Fig. 7E). Considering the structural similarity and partial redundancy of Pim kinases, we have confirmed that Pim-1, Pim-2, and Pim-3 are all up-regulated in the LPS-mediated inflammatory response (supplementary Fig. 1A–D). Moreover knockdown of both *PIM2* and *PIM3* suppressed LPS-induced upregulation of TNF-α in macrophage-like THP-1 cells (supplementary Fig. 1E). These results suggested that all Pim kinases play a similar role in the LPS-induced inflammatory response of macrophage-like THP-1 cells. To determine the role of Pim in the inflammatory response of T cells, Jurkat T cells were activated via treatment with anti-CD3/CD28. Knockdown of both *PIM1* and *PIM3* suppressed the anti-CD3/CD28-stimulated upregulation of NFATc-1 and IL-2 in Jurkat T cells (supplementary Fig. 2A–D) However, *PIM2* knockdown enhanced the anti-CD3/CD28-stimulated upregulation of NFATc-1 and IL-2 in Jurkat T cells (supplementary Fig. 2A, C). The results suggested that unlike Pim-1 and Pim-3, Pim-2 plays a role in stimulating inflammatory responses in Jurkat T cells.

## Discussion

Pim-1 kinase is a proto-oncogene encoding a serine/threonine kinase [35], which is constitutively activated when expressed [32]. The Pim-1 gene is located on chromosome 17 in humans and chromosome 6 in mice [5]. Pim-1 has been mainly studied in the field of cancer biology and in the contexts of cell cycle regulation, survival, cell growth, and senescence [36]. Recently, more research has focused on the function of Pim-1 in relation to inflammation. The inhibition of Pim-1 in an LPS-induced acute lung injury model reduces cytokine and p65 upregulation [37]. Further, Pim-1 regulates the activities of transcription factors such as NFATc1 and NF-κB [27, 38, 39]. NFATc1 and NF-κB play important roles in inflammatory responses, and the former plays an important role in regulating Th1 and Th2 patterns in cytokine production [40, 41]. Moreover, PIM1 is upregulated in RA synovial tissues and RA-FLS, and its inhibition significantly reduces the proliferation, migration, and matrix metalloproteinase production in RA-FLS in vitro [16]. These findings suggest that PIM1 kinase represents a potential therapeutic target for the regulation of inflammation. Therefore, investigating the mechanisms and functions of PIM kinase in inflammation regulation is likely to improve the prognosis of patients with inflammatory diseases. However, the mechanisms via which PIM1 regulates LPS-induced inflammation and its association with the NLRP3 inflammasome remain unclear. In the present study, the potential of PIM1 as a target for inflammation regulation was evaluated in various immune cells including macrophage-like THP-1 cells, RAW 264.7 cells, BV2 cells, and Jurkat T cells. The pan-PIM kinase inhibitor PIM447 inhibits the LPS-induced upregulation of *IL1B* in RAW 264.7 cells [14]. In the present study, the two pan-PIM kinase inhibitors PIM447 and AZD1208 suppressed the LPS-induced upregulation of Pim-1, p-Bad (Ser112), and the pro-inflammatory cytokines, pro-IL-1β, TNF-α, and IL-6 (Fig. 1G, H); activation of NF-κB (Fig. 3H); and activation of the NLRP3 inflammasome (Fig. 4G) in macrophage-like THP-1 cells. Additionally, a Pim-1 specific inhibitor, SMI-4a inhibited LPS-mediated upregulation of pro-IL-1β and IL-6 in macrophage-like THP-1 cells (Supplementary Fig. 1F). These results suggested that Pim-1 kinase plays an important role in the LPS-mediated inflammatory signaling pathway.

*Pim-1* siRNA transfection was performed to investigate the mechanisms underlying the effects of PIM1, which is most prominently studied in various inflammatory disease models [13, 15, 42–45]. We previously demonstrated that LPS treatment increases Pim-1 protein expression, and *Pim-1* knockdown suppresses the LPS-mediated upregulation of pro-IL-1β and p-Bad (Ser112) in RAW 264.7 cells [14]. Similarly, *PIM1* knockdown inhibited the LPS-induced upregulation of *PIM1*, *IL1B*, *TNFα*, and *IL6* and the increase in protein levels of pro-IL-1β, TNF-α, IL-6, Pim-1, and p-Bad (Ser112) in macrophage-like THP-1 cells in the present study (Fig. 2). iNOS, which is important for nitric oxide production associated with various pathophysiological conditions [46], and COX-2, a target for controlling inflammation with relatively fewer side effects such as gastrointestinal irritation [47], are key mediators in the inflammatory response. *PIM1* knockdown attenuated the LPS-induced increase in iNOS and COX-2 protein and mRNA expression levels (Fig. 3A–C). TLRs engage several intracellular molecules, such as NF-κB, MAPKs, and the NLRP3 inflammasome, which regulate the inflammatory response [22, 23]. The transcription factor NF-κB regulates genes involved in inflammatory responses [48]. An IKK complex comprising the subunits IKKα, IKKβ, and IKKγ regulates the activity of IκB to inhibit NF-κB and functions as a key factor for NF-κB activation [49]. *PIM1* knockdown suppressed p-IKKα/β and p-NF-κB p65 function (Fig. 3F). In addition, *PIM1* knockdown inhibited the translocation of NF-κB p65 from the cytoplasm to the nucleus in LPS-treated macrophage-like THP-1 cells. Notably, LPS treatment induced the phosphorylation of NF-κB p65 at Ser536, which was inhibited by *PIM1* knockdown. The Ser536 residue of the p65 subunit responds to inflammatory stimuli and contains the most potent phosphorylation sites, which are highly conserved in other species, and it potentially plays a role in NF-κB activity regulation [50]. The phosphorylation of RelA/p65 at Ser276 is activated when Pim-1 is stimulated by TNF-α [39]. Unlike the phosphorylation of Ser276 of p65, which is an essential contributor to both endogenous and external activation of NF-κB [51], the functional contribution of Ser536 to IKK-mediated phosphorylation remains unknown [52]. Therefore, further studies on the correlation between LPS-induced upregulation of Pim-1 and phosphorylation of NF-κB p65 at Ser276 are needed. MAPKs play an important role in the production and downstream signaling of inflammatory cytokines [53]. *PIM1* knockdown inhibited the phosphorylation of ERK, JNK and p38 in the MAPK pathway. These results indicated that Pim-1 regulates the LPS-mediated inflammation in the upstream of NF-κB p65 and MAPKs.

We determined the effects of Pim-1 on the activity of inflammasomes, a complex of inflammatory molecules, in the LPS-mediated inflammatory signaling pathway. Activation of the NLRP3 inflammasome has been extensively studied in the context of maturation and secretion of IL-1β, and it is involved in initial responses to inflammation [54]. The NLRP3 inflammasome induces the proteolytic cleavage of pro-caspase-1 into active caspase-1, which converts the cytokine precursor pro-IL-1β into mature and biologically active IL-1β [55]. In the present study, *PIM1* knockdown suppressed NLRP3 and pro-IL-1β expression, caspase-1 activation, and IL-1β secretion. Caspase-11 plays an important role in the non-canonical NLRP3 inflammasome signaling pathway [56]. However, *Pim1* knockdown does not affect the expression levels of caspase-11 protein (Fig. 4C). These results suggested that LPS-mediated Pim-1 upregulation plays a critical role in the canonical NLRP3 inflammasome signaling pathway. In this study, *PIM1* knockdown did not alter the TLR4 and adapter protein MyD88 expression levels in LPS-treated macrophage-like THP-1 cells. TAK1 functions as a major factor that promotes inflammatory pathways, triggering LPS-mediated TLR4 to initiate downstream signaling cascades [57]. TAK1 was originally identified as an MAP3K that is activated by transforming growth factor-β, but was later characterized as a major regulator of inflammation and immune signals mediated by cytokines, TLR, and T and B cell receptors [58, 59]. Additionally, TAK1 activates the IκB kinase complex and phosphorylates NF-κB to activate the NF-κB pathway [60].

*PIM1* knockdown suppressed the phosphorylation of TAK1 at Ser412, and treatment with the TAK1 inhibitor (5Z)-7-oxozeaenol inhibited the LPS-mediated upregulation of Pim-1 (Fig. 5C–E). In contrast, treatment with the pan-PIM kinase inhibitors inhibited the LPS-mediated phosphorylation of TAK1. Pim-1 kinase interacts with several proteins participating in various signaling pathways [5, 61]. The kinase domain of Pim-1 is important for the interactions of TAK1 and Pim-1 bonds in the overexpressed states [38]. In the present study, endogenous Pim-1 interacted with TAK1 in non-treated macrophage-like THP-1 cells. Moreover, LPS treatment further increased the binding of Pim-1 and TAK1 (Fig. 5F). Additionally, *PIM1* knockdown and (5Z)-7-oxozeaenol treatment inhibited the LPS-enhanced binding of Pim-1 and TAK1 (Fig. 5G, H). These results demonstrated that the interaction between Pim-1 and TAK1 plays an important role in the LPS-mediated inflammatory response signaling pathway. Pim1*-*DN and Pim1*-*K67M inhibited the LPS-mediated interaction of Pim-1 and TAK1 in both RAW 264.7 and BV2 cells (Fig. 5I, J). These results suggested that the kinase domain of Pim-1 plays an important role in interactions with TAK-1. The JAK/STAT pathway, which is an important therapeutic target in inflammatory diseases [62], regulates Pim-1 via transcriptional regulation [10, 35]. Secretion of pro-inflammatory cytokines by the LPS/TLR4/NF-κB pathway lead to activation of the JAK/STAT signaling pathway [25]. Additionally, PIM1 downregulates the JAK/STAT pathway [63]. In the present study, *PIM1* knockdown inhibited LPS-induced phosphorylation of JAK1 and STAT3 in macrophage-like THP-1 cells (Fig. 6). These results suggested that Pim-1 may positively regulate the JAK/STAT pathway in the LPS-mediated inflammatory signaling pathway. We investigated the role of the three isozymes of Pim kinase in inflammatory signaling pathways using THP-1 and Jurkat T cells. All Pim kinases functioned as positive regulators in the inflammatory response of macrophage-like THP-1 cells (Fig. 7A–E). However, we found that in contrast to Pim-1 and Pim-3, Pim-2 functioned as a negative regulator in activated Jurkat T cells (supplementary Fig. 2A–D). These results are similar to previous findings showing that Pim-2 functions as a negative regulator in T cell immune responses [64].

Taken together, our results demonstrated that Pim-1 is a positive regulator of LPS-mediated inflammatory signal transduction in macrophages via the activation of NF-κB, MAPKs, and the NLRP3 inflammasome and via the interaction of Pim-1 and TAK1. Furthermore, Pim-2 may function as a negative regulator in the inflammatory response of T cells. Collectively, these findings suggest that targeting Pim-1 could be beneficial for the treatment of inflammatory diseases. Additionally, it is necessary to modulate Pim isoforms individually to regulate the inflammatory response.

## Electronic supplementary material

Below is the link to the electronic supplementary material.

Supplementary Material 1

Supplementary Material 2

## Data Availability

The data that support the findings of this study are available from the corresponding author upon reasonable request.
