# Supplementary material for: The role of Pim-1 kinases in inflammatory signaling pathways
Source: Inflamm Res. 2024 Jul 30;73(10):1671–85. doi: 10.1007/s00011-024-01924-2 (PMC11457682; doi:10.1007/s00011-024-01924-2)
Supplement: Supplementary file 1 — Supplementary Material 1 [file 11_2024_1924_MOESM1_ESM.docx]

Table 1. Primer Sequences

| Primers | Sequences (5’→ 3’) | |
| --- | --- | --- |
| Human primer | | |
| *PIM1* | Forward | GCT CGG TCT ACT CAG GCA TC |
|  | Reverse | CGG GCA TCT GAC AAG AGA GG |
| *PIM2* | Forward | GCC TCA CAG ATC GAC TCC AG |
|  | Reverse | GAA GCA GGG CAC CAG AAC C |
| *PIM3* | Forward | ACC GCG ACA TTA AGG ACG AAA |
|  | Reverse | ACA CAC CAT ATC GTA GAG AAG CA |
| *NLRP3* | Forward | GAG GCA ACA CTC TCG GAG AC |
|  | Reverse | TCT GGC TGG AGG TCA GAA GT |
| *iNOS* | Forward | CTG TCT GGT TCC TAC GTC ACC |
|  | Reverse | CCC ACG TTA CAT GGG AGG ATA |
| *COX2* | Forward | ATC ACA GGC TTC CAT TGA CC |
|  | Reverse | TAT CAT CTA GTC CGG AGG GG |
| *IL1B* | Forward | CCT TGG GCC TCA AGG AAA A |
|  | Reverse | CTC CAG CTG TAG AGT GGG CTT A |
| *TNFα* | Forward | GGA GAA GGG TGA CCG ACT CA |
|  | Reverse | CTG CCC AGA CTC GGC AA |
| *IL6* | Forward | ATG GCA CAG TAT CTG GAG GAG |
|  | Reverse | TAA GCT GGA CTC ACT CTC GGA |

| β-actin | Forward | AAT CTG GCA CCA CAC CTT CTA |
| --- | --- | --- |
|  | Reverse | ATA GCA CAG CCt GGA TAG CAA |
