# Supplementary material for: The role of Pim-1 kinases in inflammatory signaling pathways
Source: Inflamm Res. 2024 Jul 30;73(10):1671–85. doi: 10.1007/s00011-024-01924-2 (PMC11457682; doi:10.1007/s00011-024-01924-2)
Supplement: Supplementary file 2 — Supplementary Material 2 [file 11_2024_1924_MOESM2_ESM.docx]

**Supplementary**

**Materials and Methods**

1. Electrotransfection

Jurkat T cells were seeded on 1 × 10^6^ cells / wells, and transfected with *PIM1*, *PIM2*, and *PIM3* small-interfering RNA (siRNA) (Life Technologies, Gaithersburg, MD, USA), and control siRNA using transfection reagent (sc-37007, Santa Cruz Biotechnology). siRNA electroporation was performed according to the manufacturer’s instructions with the Amaxa^TM^ cell line nucleofector kit (VCA-1005, Lonza) and program Amaxa Nucleofector TM II system (Amaxa Biosystems, Cologne, Germany)

2. RNA isolation and reverse transcription-quantitative polymerase chain reaction

Total RNA was extracted from each sample using TRIzol solution (Invitrogen, San Diego, CA, USA) according to the manufacturer's protocol. Subsequently, 1 μg of the extracted and quantified RNA was reverse transcribed using deoxynucleoside triphosphate, buffer, dithiothreitol, RNase inhibitor, and SuperScript II reverse transcriptase. The synthesized cDNA was used for reverse transcription-quantitative polymerase chain reaction performed using specific primers. The sequences were as follows: *CD80*: forward 5’-AAACTCGCATCTACT

GGCAAA-3’, reverse 5’-GGTTCTTGTACTCGGGCCATA-3’; *CD86*: forward 5’- CTGCTCATCTATACACGGTTACC-3’, reverse 5’-GGAAACGTCGTACAGTTCTGTG-3’, *CD163:* forward 5’-TTTGGACAAGCCGTGACTAGA-3’, reverse 5’-CATTCCCGGTGT

TGACATTCC-3’.


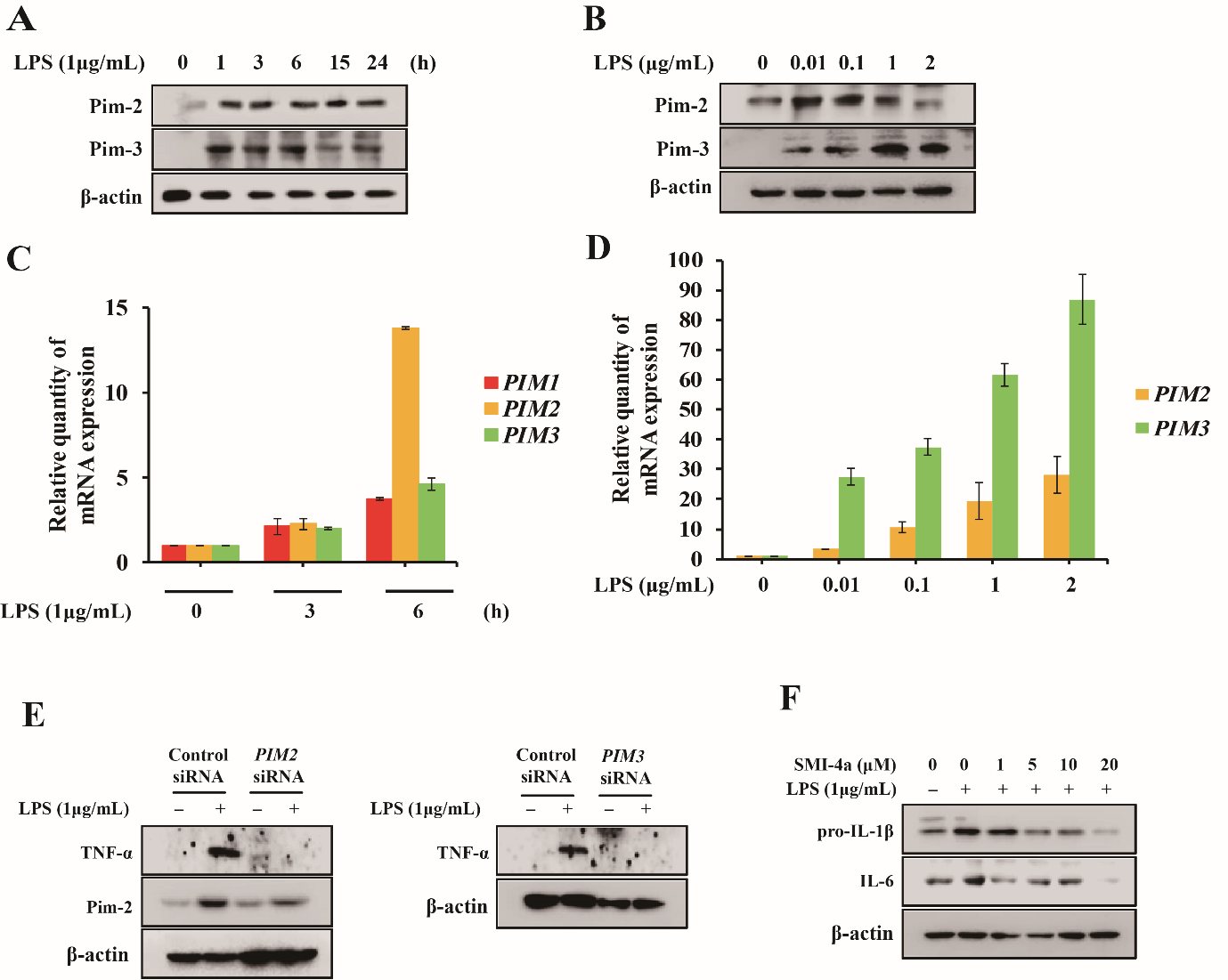


**Supplementary Fig. 1.**

(A) THP-1 cells were stimulated with LPS (1 μg/mL) for the indicated time points. Whole cell lysates were isolated and used to measure the protein expression levels of Pim-2, Pim-3 by Western blotting. (B) Cells were stimulated with LPS (0.01, 0.1, 1, and 2 μg/mL) for 6 h. Whole cell lysates were isolated and used to measure the protein expression levels of Pim-2, Pim-3 by Western blotting. (C) Cells were stimulated with LPS (1 μg/mL) for the indicated time points. Total RNA was extracted, and used to evaluate the mRNA expression levels of *PIM1, PIM2,* and *PIM3* by real-time qPCR. (D) Cells were stimulated with LPS (0.01, 0.1, 1, and 2 μg/mL) for 6 h. Total RNA was extracted, and used to evaluate the mRNA expression levels of *PIM2,* and *PIM3* by real-time qPCR. (E) THP-1 cells transfected with control siRNA or *PIM2* and *PIM3* siRNA for 72 h and then stimulated with LPS (1 μg/mL) for 6 h. Whole cell lysates were isolated and used to measure the protein expression levels of TNF-α by Western blotting. (F) Cells were pretreatment for 1h with SMI-4a (1, 5.10, and 20 μM) before stimulated with LPS (1μg/mL). Whole cell lysates were isolated and used to measure the protein expression levels of pro-IL-1β and IL-6 by Western blotting.





**Supplementary Fig. 2. The effect of Pim kinase knockdown in Jurkat T cells activation.**

(A-D) *PIM1*, *PIM2*, and *PIM3* siRNA knockdown in Jurkat T cells, respectively. And then cells were stimulted with anti-CD3/CD28 antibodies for 6 h. Whole cell lysates were isolated and used to measure the protein expression levels of NFATc-1, p-Bad and IL-2 by Western blotting.

**
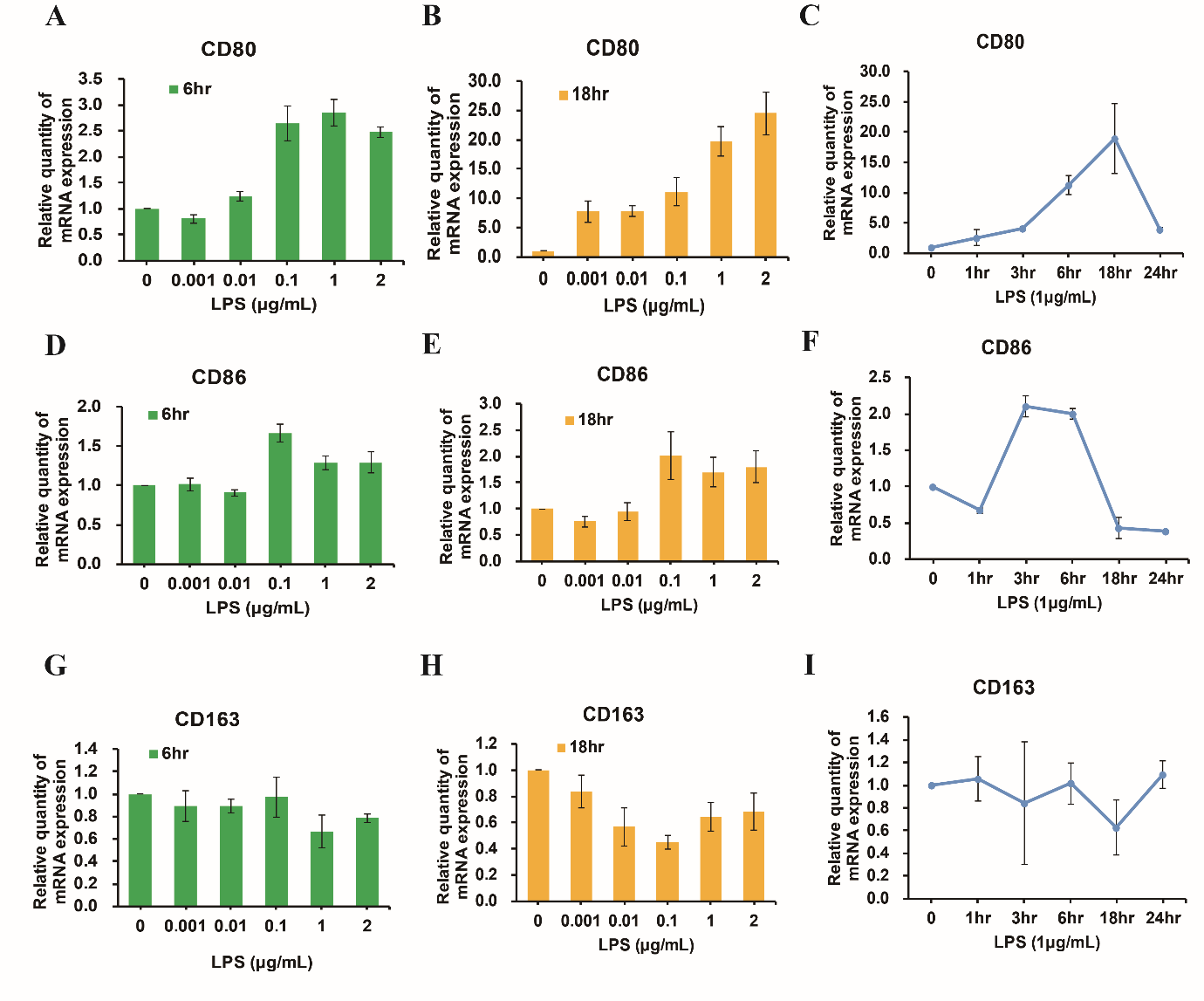
**

**Supplementary Fig. 3. M1 (CD80, CD86) and M2 (CD163) macrophage marker expression in THP-1 cells.**

(A, B, D, E, G, H) THP-1 macrophage was stimulated with LPS (0.001, 0.01, 0.1, 1, and 2μg/mL) for 6 and 28h. (C, F, I) THP-1 macrophage was stimulated with LPS (1μg/mL) for the indicated time points.
